# Herbal Medicine Containing Licorice May Be Contraindicated for a Patient with an *HSD11B2* Mutation

**DOI:** 10.1093/ecam/nep211

**Published:** 2011-02-20

**Authors:** Indra Sari Kusuma Harahap, Naoko Sasaki, Gunadi, Surini Yusoff, Myeong Jin Lee, Satoru Morikawa, Noriyuki Nishimura, Tomohiro Sasaki, Seiichiro Usuki, Midori Hirai, Mika Ohta, Yutaka Takaoka, Takashi Nishimoto, Hisahide Nishio

**Affiliations:** ^1^Department of Community Medicine and Social Healthcare Science, Kobe University Graduate School of Medicine, Kobe 650-0017, Japan; ^2^Daikakuji Clinic, Kobe University Graduate School of Medicine, Tottori, Japan; ^3^Usuki Clinic, Kobe University Graduate School of Medicine, Kobe, Japan; ^4^Department of Hospital Pharmacy, Kobe University Graduate School of Medicine, Japan; ^5^Division of Applied Genome Science and Bioinformatics, Kobe University Graduate School of Medicine, Kobe, Japan; ^6^Nishimoto Clinic, Nishinomiya, Japan

## Abstract

Licorice ingestion, as well as mutations in the *HSD11B2* gene, inhibits 11**β**-hydroxysteroid dehydrogenase type 2 (11**β**HSD2) enzyme activity, causing the syndrome of apparent mineral corticoid excess (AME). However, the combined effect of licorice ingestion and an *HSD11B2* mutation has never been reported, until now. In this study, we demonstrated that licorice ingestion can produce overt hypertension in an individual without medical history of hypertension who is heterozygous for wild-type and mutant *HSD11B2* genes. Our patient was a 51-year-old female with serious hypertension who had been taking herbal medicine containing licorice for more than one year. She was clinically diagnosed as having licorice intoxication, because she did not present with hypertension after ceasing the herbal medicine. Molecular analysis showed that she carried a missense mutation, c.40C>T, in *HSD11B2*. In conclusion, licorice ingestion is an environmental risk factor for hypertension or AME state in patients with a mutation in *HSD11B2.* Carrying a mutation in *HSD11B2* is, conversely, a genetic risk factor for licorice-induced hypertension or AME state. Herbal medicine containing licorice may, therefore, be contraindicated in patients with an *HSD11B2* mutation.

## 1. Introduction

Licorice (*Glycyrrhizae radix*) has a long history of use in herbal medicine or traditional medicine of East Asia. Licorice has been used in combination of other herbal ingredients to harmonize and improve the effects of herbal remedy. Many researchers are now studying the effects of licorice with scientific experiments on animals or cultured cells. Recently, cytoprotective effect of licorice has been reported [1, 2].

On the other hand, licorice has been known for decades to cause mineralocorticoid-like adverse effects including hypertension, edema, heart failure or hypokalemia, even though patients have low circulating levels of aldosterone [3]. Recent studies revealed that licorice inhibits the enzyme activity of 11*β*-hydroxysteroid dehydrogenase type 2 (11*β*HSD2), resulting in a pathological state similar to mineralocorticoid excess named apparent mineralocorticoid excess state (AME; [4–6]). In licorice-induced hypertension, glycyrrhetinic acid, the active substituent of licorice, inhibits 11*β*HSD2 resulting in AME state [3, 7]. Thus, it appears that licorice intoxication is a reversible pharmacological counterpart to AME syndrome, an inherited form of AME state caused by deficiency of 11*β*HSD2 enzyme activity [3].

The 11*β*HSD2 enzyme mainly converts cortisol into cortisone [3, 7, 8]. Of these two hormones, only cortisol has affinity to mineralocorticoid receptors, and thus induces mineralocorticoid effects. In several hypertensive syndromes, a decrease in 11*β*HSD2 activity has been described as a pathogenetic mechanism of increased blood pressure. Mutations in the gene *HSD11B2* coding for 11*β*HSD2 compromise 11*β*HSD2 enzyme activity, resulting in juvenile or late-onset forms of the AME syndrome [9, 10].

AME syndrome is an autosomal recessive disorder; homozygous or compound heterozygous mutations in the *HSD11B2* gene, encoding 11*β*HSD2, have been identified in patients [9–14]. Heterozygosity for wild and mutant *HSD11B2* alleles does not cause AME state or hypertension [9, 15], although some parents of AME patients are heterozygous for a mutation presented with a phenotype indistinguishable from “essential" hypertension [10].

However, the combined effect of licorice ingestion and the *HSD11B2* mutation has never been reported. Herbal medicine is very popular in Japan and many remedies available in Japan contain licorice. However, the frequency of the *HSD11B2* mutation carriers in the Japanese population is *∼*1% [16]. Thus, it is curious that *HSD11B2* mutation has not been reported among Japanese patients with licorice intoxication, and that the combined effect of licorice ingestion and the *HSD11B2* mutation has never been previously discussed.

In this study, we demonstrated that licorice ingestion can produce overt hypertension in an individual who is heterozygous for wild-type and mutant *HSD11B2* genes with no history of hypertension. This indicates a combined effect of licorice ingestion and the *HSD11B2* mutation.

## 2. Methods

### 2.1. Patient

The patient was a 51-year-old female with a two-year history of postmenopausal syndrome. Her weight and height were 49 kg and 153 cm. To improve the symptoms of postmenopausal syndrome, she had been taking an herbal medicine, Shimotu-ka-ninjin-kanzo-to, that is, four herbs (Rehmannia, Cnidium, Paeonia and Angelica) plus ginseng and licorice decoction for more than one year. Her daily ingestion of licorice was less than 1.0 g. She did not show hypertension in her medical history. Her systolic blood pressure was usually measured to be less than 130 mmHg. On April 15, 2006, she was admitted to a medical office with a heavy headache lasting several hours. On examination, her blood pressure while sitting was 190/110 mmHg. She was just instructed to rest quietly in bed at home and to stop taking the herbal medicine. Her blood pressure stabilized to within normal limits the following day. She did not take the herbal medicine after this episode and did not experience hypertension again. Although other clinical examinations apart from blood pressure measurement were not performed, it was strongly suspected that her paroxysmal hypertension was associated with licorice intoxication.

### 2.2. Molecular Analysis

Informed consent was obtained from the patient before the analysis. Genomic DNA was extracted from EDTA whole blood sample using a commercially available DNA extraction kit. Each exon of *HSD11B2* was PCR-amplified with the primer pairs reported previously [9], followed by denaturing high-performance liquid chromatography (DHPLC) screening and direct sequencing according to our previous reports [17]. DHPLC screening for the *HSD11B2* mutation, c.40C>T, was performed with primers as described below: 5′-GCTGGGCCGCCATGGAGC-3′ and 5′-CGGGACAACGCGATCCAGC-3′.

## 3. Results

### 3.1. DHPLC Screening for a Mutation in *HSD11B2*

**Figure 1:**
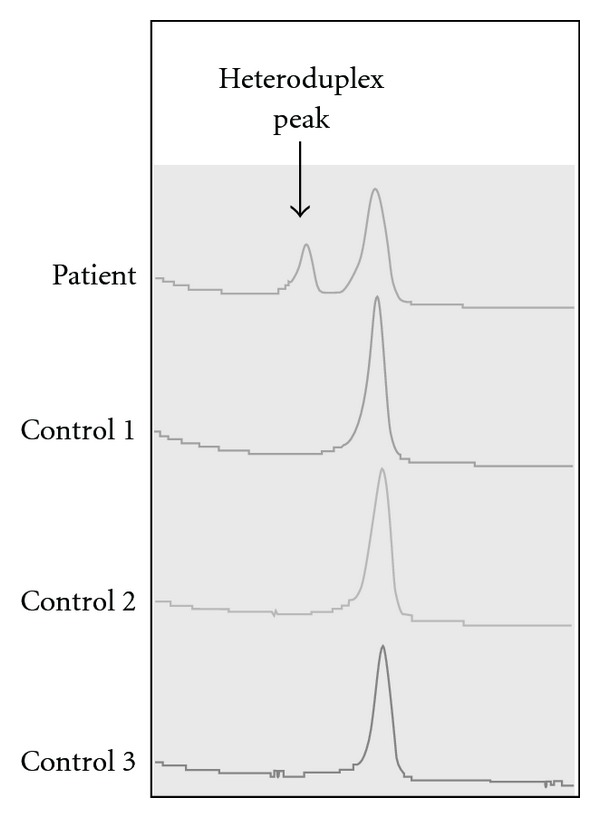
DHPLC screening for a mutation in the *HSD11B2* gene. Screening showed a small and large peak on the chromatogram of *HSD11B2* exon 1, while controls showed a single peak. The small peak indicated heteroduplex DNA formation, suggesting the presence of a mutation in the patient's *HSD11B2* exon 1.

DHPLC screening showed that the patient had a nucleotide mutation in the PCR fragment including *HSD11B2* exon 1. As shown in Figure 1, the patient had two peaks in her DHPLC chart of the PCR fragment including *HSD11B2* exon 1. This finding suggested that the patient carried nucleotide changes in this region. However, DHPLC screening did not disclose what kind of mutation was present in the patient. 

### 3.2. Identification of the Mutation in *HSD11B2*

**Figure 2:**
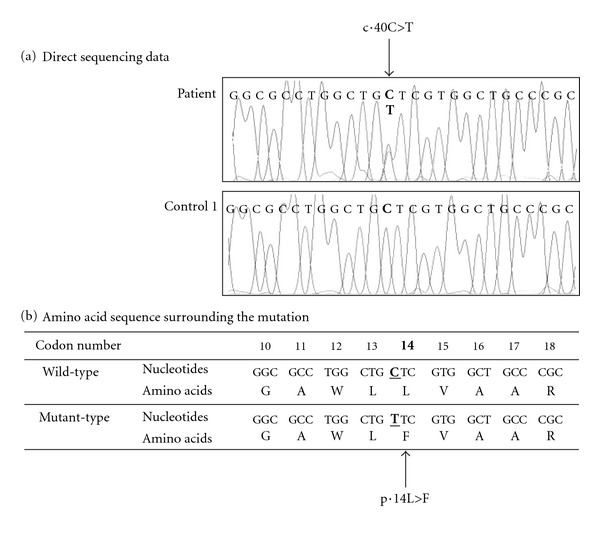
Direct DNA sequencing of *HSD11B2* exon 1 (a) and amino acid sequence surrounding the mutation (b). DNA sequencing showed heterozygous status for the wild-type (C at nucleotide position 40) and mutant (T at nucleotide position 40) sequences.

To identify the mutation in the patient, we conducted a direct DNA sequencing analysis of the PCR fragment including *HSD11B2* exon 1. As shown in Figure 2, the patient carried a missense mutation, c.40C>T, leading to an amino acid substitution from leucine to phenylalanine at position 14 of 11*β*HSD2 (p.L14F). Figure 2 also demonstrates that the patient was heterozygous for normal and mutant alleles. In other regions of the *HSD11B2* gene, no nucleotide change was observed in the patient. 

## 4. Discussion

### 4.1. Heterozygous Mutation in *HSD11B2*

We identified a heterozygous missense mutation, c.40C>T, in the *HSD11B2* gene in a Japanese patient with licorice-induced hypertension. Kamide et al. [16] first reported the c.40C>T in *HSD11B2* exon 1 in the Japanese population. They found the mutation in 11 out of 2433 hypertensive individuals and in 8 out of 2175 normotensive individuals. Although there have not been reports of enzyme activity of mutated 11*β*HSD2 nor AME patients homozygous for the mutation, this mutation occurs in the amino acid residue that is highly conserved among three different species (human, mouse and rabbit), indicating that the mutation may affect 11*β*HSD2 activity [16].

In general, heterozygosity for wild and mutant *HSD11B2* alleles does not play a critical role in the pathogenesis of AME state in most cases [9, 15]. Lavery et al. [10] noted that the mothers of AME patients heterozygous for missense mutations presented with a phenotype indistinguishable from essential hypertension. Taken together, all of these findings suggested that one copy of normal *HSD11B2* allele may guarantee enough levels of 11*β*HSD2 enzyme activity to prevent serious AME syndrome and that factors other than *HSD11B2* mutation may be required for the development of hypertension or AME in heterozygous patients.

### 4.2. Licorice Ingestion as an Environmental Risk Factor

**Figure 3:**
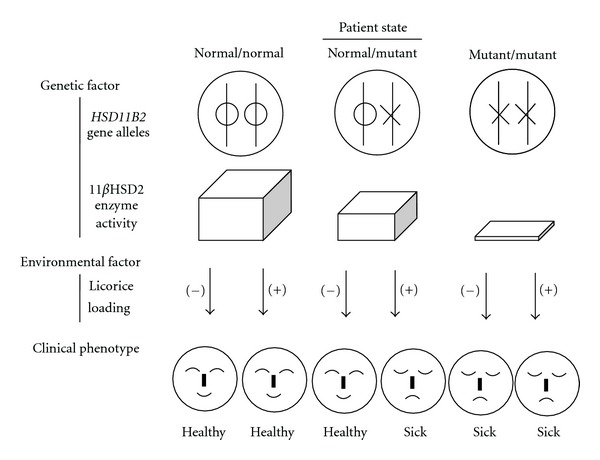
Schematic drawing of the pathogenesis of the patient. The patient was heterozygous for a mutation in *HSD11B2* (“normal/mutant" alleles in the figure), suggesting that her 11*β*HSD2 enzyme activity in the kidney was reduced. Without licorice loading, she was able to keep her healthy condition. However, with licorice loading, she showed serious hypertension.

A question then arises as to why our patient showed hypertension despite being heterozygous for an *HSD11B2* mutation. The most likely explanation is that the licorice in the herbal medicine inhibited the residual enzyme activity of normal 11*β*HSD2 in the patient, causing paroxysmal heavy headache as a symptom of AME state. Thus, we can speculate that licorice ingestion contributed to the development of hypertension in this patient (Figure 3). 

### 4.3. Carrying a Mutation in HSD11B2 as a Genetic Risk Factor

It is also noteworthy that our patient had been ingesting no more than 1.0 g of licorice per day for only one year before the paroxysmal hypertension. Yasue et al. [18] reported that a 93-year-old hypertensive female, taking herbal medicines containing licorice, suffered from severe hypokalemia, rhabdomyolysis, muscle paralysis and respiratory impairment due to a serious AME state. She had been ingesting 5.0 g of licorice daily for the last seven years. Gerritsen et al. [19] also reported that a 44-year-old previously healthy female, was admitted to the emergency department following resuscitation from ventricular fibrillation. She had been ingesting 250–500 g of licorice daily for the several years. Compared with these patients, our patient suffered from licorice intoxication after consuming a very small amount of licorice, which suggested that the presence of an *HSD11B2* mutation contributed to the development of her hypertension (Figure 3).

It may be hazardous for such patients with an *HSD11B2* mutation to take herbal medicine containing licorice. To avoid serious side effects of licorice, pharmacogenetic approaches are required. It has been reported that an A-to-G change at nucleotide 1555 in mitochondrion DNA is associated with aminoglycoside-induced hearing loss [20]. Now, genetic testing of mitochondrion DNA is widely used to avoid hearing loss caused by aminoglycosides [21]. Similarly, genetic testing of *HSD11B2* will be useful to avoid licorice-induced hypertension or AME state.

## 5. Conclusion

We applied a pharmacogenetic approach to licorice intoxication and were the first to identify the combined effect of licorice ingestion and an *HSD11B2* mutation. Licorice ingestion is an environmental risk factor for hypertension or AME state in patients with a mutation in *HSD11B2.* Carrying a mutation in *HSD11B2* is therefore, conversely, a genetic risk factor for licorice-induced hypertension or AME state. Herbal medicine containing licorice may be contraindicated for patients with an *HSD11B2* mutation. Genetic testing to detect individuals with an *HSD11B2* mutation will be useful to avoid licorice-induced adverse effects.
